# Structure-based design of prefusion-stabilized SARS-CoV-2 spikes

**DOI:** 10.1126/science.abd0826

**Published:** 2020-07-23

**Authors:** Ching-Lin Hsieh, Jory A. Goldsmith, Jeffrey M. Schaub, Andrea M. DiVenere, Hung-Che Kuo, Kamyab Javanmardi, Kevin C. Le, Daniel Wrapp, Alison G. Lee, Yutong Liu, Chia-Wei Chou, Patrick O. Byrne, Christy K. Hjorth, Nicole V. Johnson, John Ludes-Meyers, Annalee W. Nguyen, Juyeon Park, Nianshuang Wang, Dzifa Amengor, Jason J. Lavinder, Gregory C. Ippolito, Jennifer A. Maynard, Ilya J. Finkelstein, Jason S. McLellan

**Affiliations:** 1 Department of Molecular Biosciences, University of Texas, Austin, TX 78712, USA.; 2 Department of Chemical Engineering, University of Texas, Austin, TX 78712, USA.; 3 Department of Oncology, Dell Medical School, University of Texas, Austin, TX 78712, USA.; 4 Center for Systems and Synthetic Biology, University of Texas, Austin, TX 78712, USA.

## Abstract

The COVID-19 pandemic has led to accelerated efforts to develop therapeutics and vaccines. A key target of these efforts is the spike (S) protein, which is metastable and difficult to produce recombinantly. Here, we characterized 100 structure-guided spike designs and identified 26 individual substitutions that increased protein yields and stability. Testing combinations of beneficial substitutions resulted in the identification of HexaPro, a variant with six beneficial proline substitutions exhibiting ~10-fold higher expression than its parental construct and the ability to withstand heat stress, storage at room temperature, and three freeze-thaw cycles. A 3.2 Å-resolution cryo-EM structure of HexaPro confirmed that it retains the prefusion spike conformation. High-yield production of a stabilized prefusion spike protein will accelerate the development of vaccines and serological diagnostics for SARS-CoV-2.

SARS-CoV-2 is a novel betacoronavirus that emerged in Wuhan, China in December 2019 and is the causative agent of the COVID-19 pandemic (*1*, *2*). Effective vaccines, therapeutic antibodies and small-molecule inhibitors are urgently needed, and the development of these interventions is proceeding rapidly. Coronavirus virions are decorated with a spike (S) glycoprotein that binds to host-cell receptors and mediates cell entry via fusion of the host and viral membranes (*3*). Binding of the SARS-CoV-2 spike to the angiotensin-converting enzyme 2 (ACE2) receptor (*4*–*6*) triggers a large conformational rearrangement of the spike from a metastable prefusion conformation to a highly stable postfusion conformation, facilitating membrane fusion (*7*, *8*). Attachment and entry are essential for the viral life cycle, making the S protein a primary target of neutralizing antibodies and a critical vaccine antigen (*9*, *10*).

Prefusion stabilization tends to increase the recombinant expression of viral fusion glycoproteins, possibly by preventing triggering or misfolding that results from a tendency to adopt the more stable postfusion structure. Prefusion-stabilized viral glycoproteins are also superior immunogens to their wild-type counterparts (*11*–*13*). Structure-based design of prefusion-stabilized MERS-CoV and SARS-CoV spike ectodomains resulted in homogeneous preparations of prefusion spikes and greatly increased yields (*11*). These variants (S-2P) contained two consecutive proline substitutions in the S2 subunit in a turn between the central helix and heptad repeat 1 (HR1) that must transition to a single, elongated α-helix in the postfusion conformation. These S2-P spikes have been used to determine high-resolution structures by cryo-EM (*14*–*17*), including for SARS-CoV-2 (*18*, *19*), and have accelerated development of vaccine candidates. However, even with these substitutions, the SARS-CoV-2 S-2P ectodomain is unstable and difficult to produce reliably in mammalian cells, hampering biochemical research and development of subunit vaccines.

To generate a prefusion-stabilized SARS-CoV-2 spike protein that expresses at higher levels and is more stable than our original S-2P construct (*18*) we analyzed the SARS-CoV-2 S-2P cryo-EM structure (PDB ID: 6VSB) and designed substitutions based upon knowledge of class I viral fusion protein function and general protein stability principles. These strategies included the introduction of disulfide bonds to prevent conformational changes during the pre-to-postfusion transition, salt bridges to neutralize charge imbalances, hydrophobic residues to fill internal cavities, and prolines to cap helices or stabilize loops in the prefusion state. We cloned 100 single S-2P variants and characterized their relative expression levels (table S1), and for those that expressed well we characterized their monodispersity, thermostability, and quaternary structure. Given that the S2 subunit undergoes large-scale refolding during the pre-to-postfusion transition, we exclusively focused our efforts on stabilizing S2. Substitutions of each category were identified that increased expression while maintaining the prefusion conformation (Fig. 1 and 2A). Overall, 26 out of the 100 single-substitution variants had higher expression than S-2P (table S1).

**Fig. 1:**
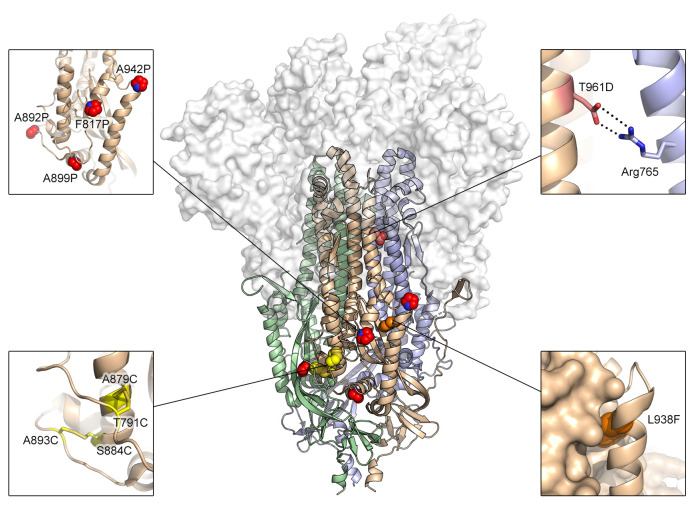
Exemplary substitutions for SARS-CoV-2 spike stabilization. Side view of the trimeric SARS-CoV-2 spike ectodomain in a prefusion conformation (PDB ID: 6VSB). The S1 domains are shown as a transparent molecular surface. The S2 domain for each protomer is shown as a ribbon diagram. Each inset corresponds to one of four types of spike modifications (proline, salt bridge, disulfide, cavity filling). Side chains in each inset are shown as red spheres (proline), yellow sticks (disulfide), red and blue sticks (salt bridge) and orange spheres (cavity filling).

**Fig. 2:**
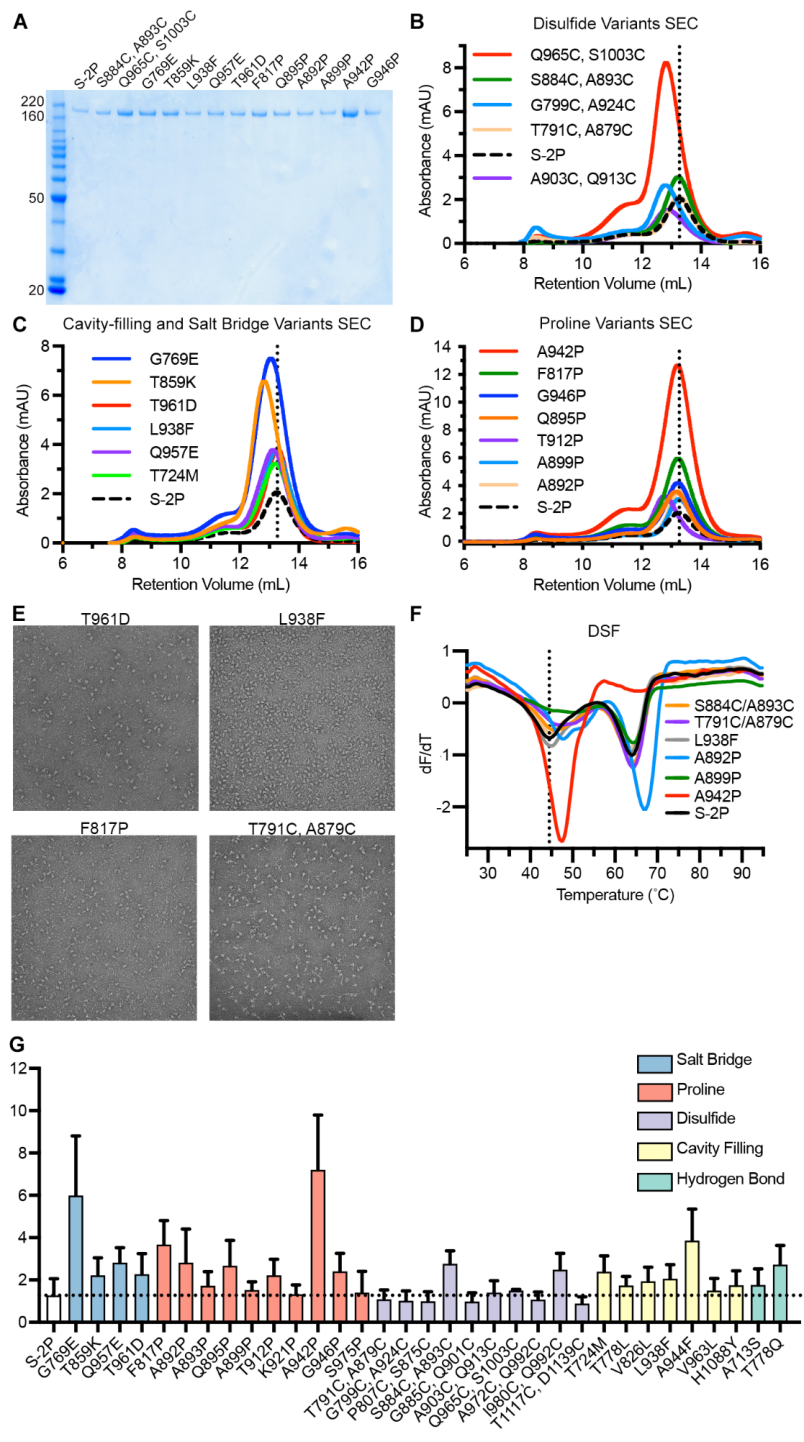
Characterization of single-substitution spike variants. (**A**) SDS-PAGE of SARS-CoV-2 S-2P and single-substitution spike variants. Molecular weight standards are indicated at the left in kDa. (**B** to **D**) Size-exclusion chromatography traces of purified spike variants, grouped by type (B, disulfide variants; C, cavity filling and salt bridge; D, proline). A vertical dotted line indicates the characteristic peak retention volume for S-2P. (**E**) Representative negative stain electron micrographs for four variants. (**F**) Differential scanning fluorimetry analysis of spike variant thermostability. The vertical dotted line indicates the first apparent melting temperature for S-2P. (**G**) Expression levels of individual variants determined by quantitative biolayer interferometry. Variants are colored by type. The horizontal dotted line indicates the calculated concentration of S-2P, which was used as a control for comparison. The mean of three biological replicates is plotted, with error bars indicating standard deviations.

One common strategy to stabilize class I fusion proteins is to covalently link a region that undergoes a conformational change to a region that does not via a disulfide bond. For instance, the Q965C/S1003C substitution aims to link HR1 to the central helix, whereas G799C/A924C aims to link HR1 to the upstream helix. These two variants boosted protein expression 3.8-fold and 1.3-fold compared to S-2P, respectively (Fig. 2B). However, the size-exclusion chromatography (SEC) traces of both variants showed a leftward shift compared to S-2P, indicating that the proteins were running larger than expected, which agreed well with negative stain electron microscopy (nsEM) results that showed partially misfolded spike particles (fig. S1). Although introduction of disulfide bonds has been successful in the case of HIV-1 Env (SOSIP) and RSV F (DS-Cav1) (*12*, *20*), it generally had detrimental effects for SARS-CoV-2 S, but there were a few exceptions. The S884C/A893C and T791C/A879C variants eluted on SEC at a volume similar to S-2P and were well-folded trimeric particles by nsEM (Fig. 2E). These variants link the same α-helix to two different flexible loops that pack against a neighboring protomer (Fig. 1). Notably, S884C/A893C had two-fold higher expression than S-2P with slightly increased thermostability (Fig. 2, F and G).

Introducing a salt bridge at the HIV-1 gp120–gp41 interface has been previously shown to boost expression and enhance the binding of trimer-specific antibodies (*21*). Based on a similar principle, the T961D and G769E substitutions were introduced to form inter-protomeric electrostatic interactions with Arg765 and Arg1014, respectively (Fig. 1). Both variants increased expression and resembled well-folded trimeric spikes (Fig. 2, C and E, fig. S2, and table S1). In addition to salt bridges, filling loosely packed hydrophobic cores that allow the protein to refold can help stabilize the prefusion state, as shown by previous cavity-filling substitutions in RSV F and HIV-1 Env (*12*, *20*, *22*). Here, the L938F substitution was designed to fill a cavity formed in part by HR1, the fusion peptide and a β-hairpin (Fig. 1). This substitution resulted in a 2-fold increase in expression (Fig. 2C) that was additive in combination with disulfide or proline substitutions (table S2).

Previous successes using proline substitutions inspired us to investigate 14 individual variants wherein a proline was substituted into flexible loops or the N-termini of helices in the fusion peptide, HR1, and the region connecting them (CR) (Fig. 2, D and G, and table S1). As expected, multiple proline variants boosted the protein expression and increased the thermostability (Fig. 2, D, F, and G). Two of the most successful substitutions, F817P and A942P, exhibited 2.8 and 6.0-fold increases in protein yield relative to S-2P, respectively. The A942P substitution further increased the melting temperature (Tm) by ~3°C, and both variants appeared as well-folded trimers by nsEM (Fig. 2E and fig. S2). This result is reminiscent of previous successful applications of proline substitutions to class I fusion proteins including HIV-1 Env, influenza HA, RSV F, hMPV F, MERS-CoV S, Lassa GPC and Ebola GP (*11*, *12*, *22*–*26*).

We next generated combination (“Combo”) variants that combined the best-performing substitutions from our initial screen. The Combo variants containing two disulfide bonds generally expressed 2-fold lower than the single-disulfide variants, suggesting that they interfered with each other (table S2). Adding one disulfide (S884C/A893C) to a single proline variant (F817P) also reduced the expression level, although the quaternary structure of the spikes was well maintained (table S2, Combo40). The beneficial effect of a disulfide bond was most prominent when combined with L938F, a cavity-filling variant. Combo23 (S884C/A893C, L938F) had higher protein yields than either of its parental variants, but the Tm of Combo23 did not increase compared to S884C/A893C (fig. S3). In addition, mixing one cavity-filling substitution with one proline substitution (Combo20) increased the expression compared to L938F alone (table S2).

Combining multiple proline substitutions resulted in the most substantial increases in expression and stability (Fig. 3A). Combo14, containing A892P and A942P, had a 6.2-fold increase in protein yield compared to A892P alone (Fig. 3B). Adding a third proline, A899P (Combo45), increased thermostability (+1.2°C Tm) but did not further increase expression (Fig. 3C). Combo46 (A892P, A899P, F817P) had a 3.4-fold increase in protein yield and a 3.3°C rise in Tm as compared to A892P. The most promising variant, Combo47, renamed HexaPro, contains all four beneficial proline substitutions (F817P, A892P, A899P, A942P) as well as the two proline substitutions in S-2P. HexaPro expressed 9.8-fold higher than S-2P, had ~5°C increase in Tm, and retained the trimeric prefusion conformation (Fig. 3D). We focused on this construct for additional characterization.

**Fig. 3:**
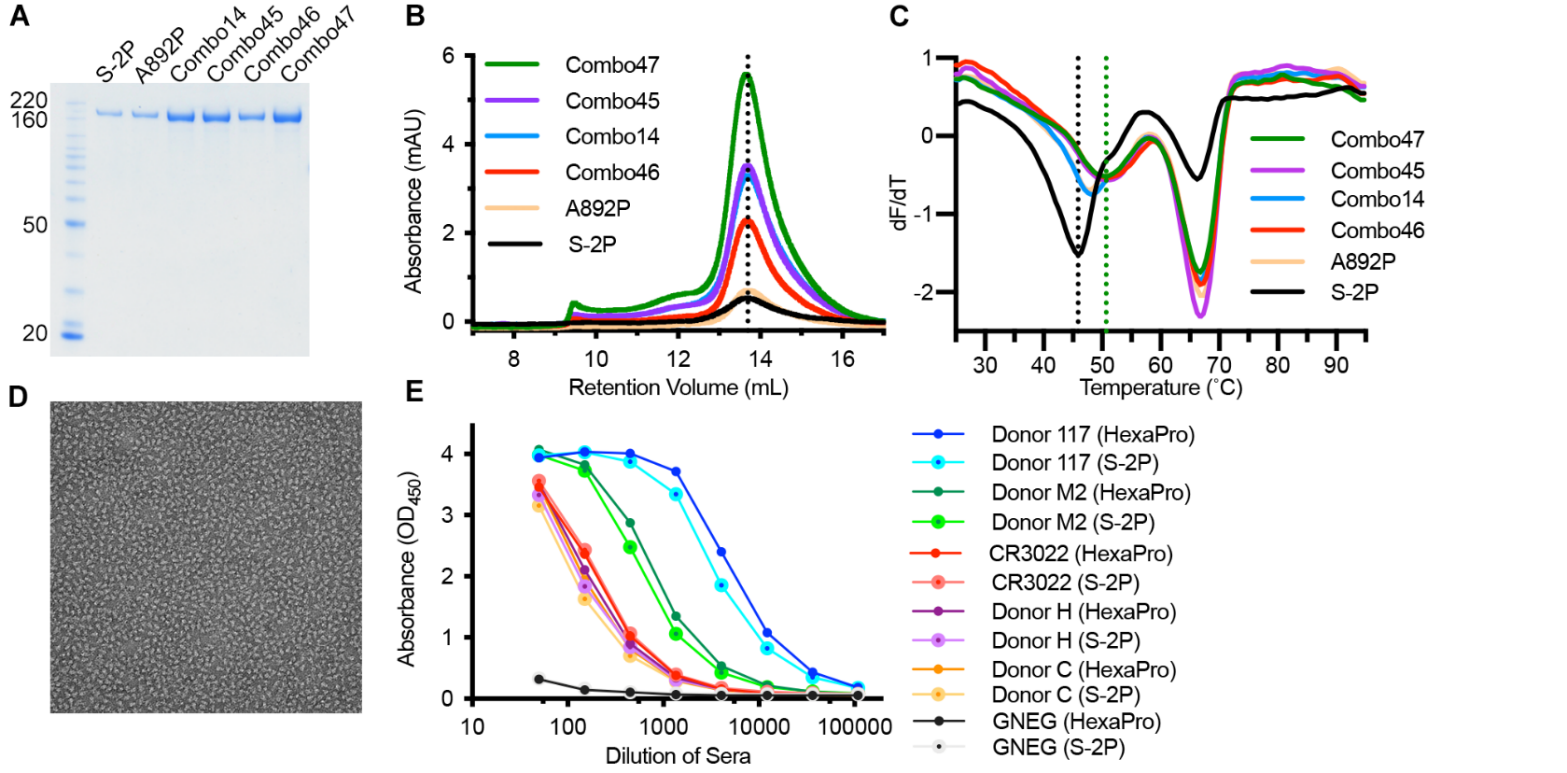
**Characterization of multi-substitution spike variants**. (**A**) SDS-PAGE of SARS-CoV-2 Combo variants. Molecular weight standards are indicated at the left in kDa. (**B**) SEC traces for S-2P, A892P and four Combo variants. The vertical dotted line indicates the peak retention volume for S-2P. (**C**) DSF analysis of Combo variant thermostability. The black vertical dotted line indicates the first apparent melting temperature for S-2P and the green vertical dotted line indicates the first apparent melting temperature for Combo47 (HexaPro). (**D**) Negative stain electron micrograph of purified Combo47 (HexaPro). (**E**) Binding of S-2P or HexaPro to convalescent human sera, mAb CR3022 and negative control serum (GNEG), measured by ELISA.

To assess the viability of HexaPro as a potential vaccine antigen or diagnostic reagent, we comprehensively examined large-scale production in FreeStyle 293-F cells, feasibility of protein expression in ExpiCHO cells, epitope integrity and protein stability. We were able to generate ~21 mg of HexaPro from 2L of FreeStyle 293-F cells, or 10.5 mg/L, which represents a greater than 10-fold improvement over S-2P (*18*). Large-scale HexaPro preparations retained a monodisperse SEC peak corresponding to the molecular weight of a glycosylated trimer (fig. S4A) and were indistinguishable from S-2P by nsEM (fig. S4B). Industrial production of recombinant proteins typically relies on CHO cells rather than HEK293 cells. We thus investigated HexaPro expression in ExpiCHO cells via transient transfection. ExpiCHO cells produced 1.3 mg of well-folded protein per 40 mL of culture, or 32.5 mg/L (fig. S4, C and D). In addition, the binding kinetics of HexaPro to the human ACE2 receptor were comparable to those of S-2P (fig. S4, E and F), with affinities of 13.3 nM and 11.3 nM, respectively. HexaPro remained folded in the prefusion conformation after 3 cycles of freeze-thaw, 2 days incubation at room temperature or 30 min at 55°C (fig. S4, G and H). In contrast, S-2P showed signs of aggregation after 3 cycles of freeze-thaw and began unfolding after 30 min at 50°C. Importantly, HexaPro reacted to human convalescent sera and RBD-specific mAb (CR3022) (*27*) similarly to S-2P, suggesting the antigenicity of HexaPro is well-preserved (Fig. 3E). Collectively, these data indicate that HexaPro is a promising candidate for SARS-CoV-2 vaccine and diagnostic development.

To confirm that the stabilizing substitutions did not lead to any unintended conformational changes, we determined the cryo-EM structure of SARS-CoV-2 S HexaPro. From a single dataset, we were able to obtain high-resolution 3D reconstructions for two distinct conformations of S: one with a single RBD in the up conformation and the other with two RBDs in the up conformation. This two-RBD-up conformation was not observed during previous structural characterization of SARS-CoV-2 S-2P (*18*, *19*). While it is tempting to speculate that the enhanced stability of S2 in HexaPro allowed us to observe this less stable intermediate, validating this hypothesis will require further investigation. Roughly a third (30.6%) of the particles were in the two-RBD-up conformation, leading to a 3.20 Å reconstruction. The remaining particles were captured in the one-RBD-up conformation, although some flexibility in the position of the receptor-accessible RBD prompted us to remove a subset of one-RBD-up particles that lacked clear density for this domain, resulting in a final set of 85,675 particles that led to a 3.21 Å reconstruction (Fig. 4A and figs. S5 and S6). Comparison of our one-RBD-up HexaPro structure with the previously determined 3.46 Å S-2P structure revealed an RMSD of 1.2 Å over 436 Cα atoms in S2 (Fig. 4B). The relatively high resolution of this reconstruction allowed us to confirm that the stabilizing proline substitutions did not distort the S2 subunit conformation (Fig. 4C).

**Fig. 4:**
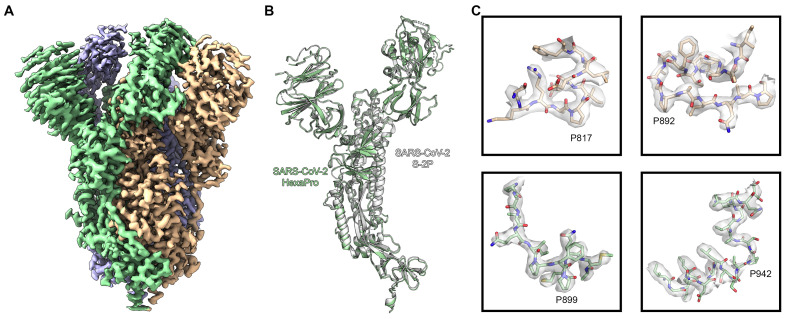
**High-resolution cryo-EM structure of HexaPro**. (**A**) EM density map of trimeric HexaPro. Each protomer is shown in a different color; the protomer depicted in wheat adopts the RBD-up conformation. (**B**) Alignment of an RBD-down protomer from HexaPro (green ribbon) with an RBD-down protomer from S-2P (white ribbon, PDB ID: 6VSB). (**C**) Zoomed view of the four proline substitutions unique to HexaPro. The EM density map is shown as a transparent surface and individual atoms are shown as sticks. Nitrogen atoms are colored blue and oxygen atoms are colored red.

The high yield and enhanced stability of HexaPro should enable industrial production of subunit vaccines and could also improve DNA or mRNA-based vaccines by producing more antigen per nucleic acid molecule, thus improving efficacy at the same dose or maintaining efficacy at lower doses. It is our hope that this work will accelerate the production of prefusion spikes to mitigate the public health emergency and has broad implications for next-generation coronavirus vaccine design.

## Supplementary Materials

science.sciencemag.org/cgi/content/full/science.abd0826/DC1 Materials and Methods Figs. S1 to S6 Tables S1 to S3 References (*28*–*33*)
